# Association of Augmentation Index With Cerebral Small Vessel Disease: A Systematic Review and Meta-Analysis

**DOI:** 10.1093/ajh/hpaf054

**Published:** 2025-04-19

**Authors:** Fotios Karachalias, Lazaros K Yofoglu, Nikolaos Kakaletsis, Konstantinos Grammatopoulos, Antonios A Argyris, Eleni Korompoki, Elpida Athanasopoulou, Theodoros G Papaioannou, Athanasios D Protogerou

**Affiliations:** Cardiovascular Prevention and Research Unit, Clinic/Laboratory of Pathophysiology, Medical School, National and Kapodistrian University of Athens, Athens, Greece; Cardiovascular Prevention and Research Unit, Clinic/Laboratory of Pathophysiology, Medical School, National and Kapodistrian University of Athens, Athens, Greece; Second Medical Department, Aristotle University of Thessaloniki, Thessaloniki, Greece; Cardiovascular Prevention and Research Unit, Clinic/Laboratory of Pathophysiology, Medical School, National and Kapodistrian University of Athens, Athens, Greece; Cardiovascular Prevention and Research Unit, Clinic/Laboratory of Pathophysiology, Medical School, National and Kapodistrian University of Athens, Athens, Greece; Department of Clinical Therapeutics, School of Medicine, National and Kapodistrian University of Athens, Athens, Greece; Department of Clinical Therapeutics, School of Medicine, National and Kapodistrian University of Athens, Athens, Greece; Divison of Brain Sciences, Imperial College London, London, United Kingdom; Cardiovascular Prevention and Research Unit, Clinic/Laboratory of Pathophysiology, Medical School, National and Kapodistrian University of Athens, Athens, Greece; Biomedical Engineering Unit, 1st Department of Cardiology, “Hippokration” Hospital, Medical School of the National and Kapodistrian University of Athens, Greece; Cardiovascular Prevention and Research Unit, Clinic/Laboratory of Pathophysiology, Medical School, National and Kapodistrian University of Athens, Athens, Greece

**Keywords:** augmentation index (AIx), blood pressure, cerebral microbleeds (CMB), cerebral small vessel disease (cSVD), hypertension, lacunes, perivascular spaces (PVS), pressure wave reflections (PWRs), pulse wave analysis (PWA), white matter hyperintensities (WMH)

## Abstract

**BACKGROUND:**

Augmentation index (Aix) is a widely used index of pressure wave reflections (PWRs) derived from noninvasive blood pressure waves recordings and pulse wave analysis. Cerebral small vessel disease (cSVD) comprises various pathological processes affecting the brain’s microvasculature and is a major cause of vascular cognitive impairment, ischemic, and hemorrhagic stroke. This meta-analysis aimed to assess the association between PWRs, as measured by AIx, and cSVD as identified through neuroimaging biomarkers, and to explore potential differences based on arterial site of AIx measurement.

**METHODS:**

Following a predefined protocol adhering to the Meta-Analysis of Observational Studies in Epidemiology guidelines, nine studies comprising a total of 6,774 participants were included. Pooled effect estimates were calculated to evaluate the relationship between AIx and cSVD markers, including white matter hyperintensities (WMH).

**RESULTS:**

A marginal positive association was observed between AIx and cSVD [central (carotid and aortic) AIx: β=0.04 (95% CI: 0.01-0.07) per SD increase; carotid AIx: β=0.05 (95% CI: 0.00-0.09); aortic AIx: β=0.03 (95% CI: 0.00-0.06)]. Similar results were found for the association between AIx and WMH [central (carotid and aortic) AIx: β=0.05 (95% CI: 0.01-0.08); carotid AIx: β=0.05 (95% CI: 0.00-0.10); aortic AIx: β=0.05 (95% CI: 0.00-0.010)].

**CONCLUSIONS:**

AIx showed a marginal positive association with cSVD, but the clinical relevance remains unclear. Substantial heterogeneity across studies, along with the absence of data on other PWR indices, limits the robustness and generalizability of these findings. Future research should employ advanced methodologies capable of assessing local PWR coefficients to better elucidate the relationship between PWRs and brain microcirculation.

## Graphical Abstract

**Graphical Abstract:**
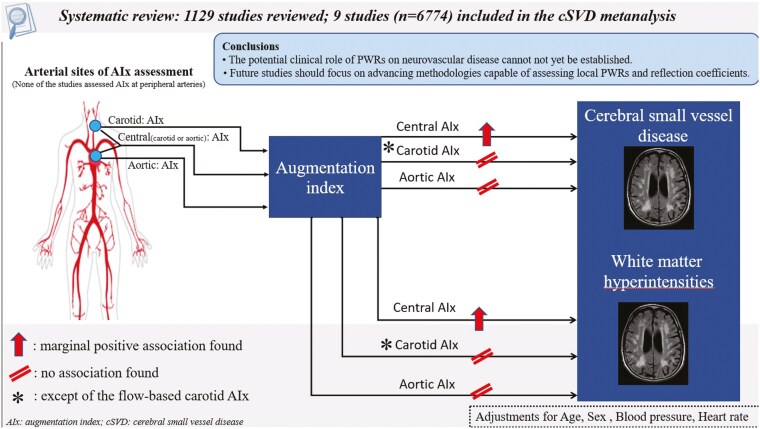

Retrograde traveling blood pressure wave reflections (PWRs) occur at various points of impedance mismatch within the arterial system and constitute an important component of cardiovascular (CV) physiology.^1,2^ Although several indices, such as the backward pressure wave (P_b_), derived from complex methodologies and potentially provide detailed information regarding the quantification and clinical value of PWRs,^1,3–5^ the augmentation index (AIx) is a commonly used, though imperfect, index of PWRs obtained through noninvasive blood pressure (BP) waves recordings and pulse wave analysis (PWA).^6,7^

Augmentation index appears to represent a modifiable CV risk factor and an intriguing therapeutic target in CV medicine, as indicated by the limited clinical trials conducted so far.^8,9^ Previous studies have shown that different classes of BP-lowering drugs and several widely consumed nutrients have variable effects on PWRs, independent of their effect on mean BP and arterial stiffness.^10–12^ Moreover, a 10% increase in the AIx, measured at the carotid artery and aorta, is associated with a 40% higher risk of all-cause mortality and a 30% higher risk of CV events, independent of BP levels.^13^ However, the exact role of PWRs in CV pathophysiology of both macro- and microcirculation remains complex and not yet fully understood.^14^

A recent meta-analysis found that higher PWRs, assessed at any arterial site proximal or distal to the heart, are associated with increased left ventricular mass (LVM).^3^ This association is thought to result from either an increased magnitude of PWRs, likely driven by vascular remodeling that raises the arteriolar reflection coefficient,^2,15^ and/or the inappropriate early systolic timing of PWRs upon their arrival at the level of the central arteries.^10,14,16–18^ On the other hand, it has been suggested—but remains controversial—that an increased magnitude of PWRs generated at the micro-macrovascular interface might protect the microcirculation from the detrimental effects of elevated flow and pressure pulsatility. However, evidence regarding its impact on the microcirculation of the brain and kidneys is conflicting.^14,19,20^

Cerebral small vessel disease (cSVD) encompasses a range of pathological processes that affect the brain’s perforating arterioles, capillaries, and venules.^21,22^ Cerebral small vessel disease is the leading cause of vascular cognitive impairment,^23^ accounting for 25% of all ischemic strokes^24^ and the majority of intracerebral hemorrhage cases^25^; moreover, it is an independent predictor of mortality.^26,27^ Various neuroimaging markers are used to assess cSVD including lacunes, white matter hyperintensities (WMH), cerebral microbleeds (CMB), and enlarged perivascular spaces (PVS).^28^ Despite its high prevalence in older populations (with a prevalence ≥ 90% in individuals aged above 65 years^29^), the underlying mechanisms of cSVD are not well understood, and the role of PWRs in cSVD and neurocognitive function is currently under investigation.^30^

The aim of this systematic review and meta-analysis was to evaluate the available evidence on the association between PWRs, as measured by the AIx, and the presence of cSVD identified through neuroimaging markers. Additionally, it sought to investigate whether this association varies based on the arterial site of PWRs assessment.

## METHODS

This systematic review adhered to a predefined protocol registered with PROSPERO (CRD42023396806, https://www.crd.york.ac.uk/PROSPERO/display_record.php?RecordID=396806) and followed the Meta-analysis of Observational Studies in Epidemiology guidelines.^31^

### Eligibility criteria

Cohort, cross-sectional, and case-control studies, along with primary or secondary analyses of randomized controlled trials investigating the association between Aix (as index of PWRs) and cSVD, were considered eligible. Studies conducted in the general population or among high-risk populations, including patients with stroke, hypertension, CV disease, diabetes mellitus, or chronic kidney disease, were included. Exclusions applied to studies with fewer than 100 participants to minimize effect size overestimation, as well as those involving children, adolescents, rare genetic disorders, in vitro or animal-based research, reviews (systematic, narrative, or meta-analyses), case reports, case series, genetic diseases predisposing to cSVD (e.g., Cerebral Autosomal Dominant/Recessive Arteriopathy with Subcortical Infarcts and Leukoencephalopathy [CADASIL, CARASIL], Fabry disease). Research focusing on autoimmune diseases, vasculitis, primary cardiomyopathies (e.g., dilatative or hypertrophic), and studies exclusively involving individuals with dementia were also excluded.

### Study selection and data extraction

Selection and data extraction were conducted independently by two reviewers (L.Y. and K.G.), who systematically searched PubMed and Scopus from inception to 31 December 2023, using predefined search terms (**Supplementary Material**). Reference lists of eligible articles were manually reviewed to identify additional relevant studies not captured in the initial search (“snowball” procedure). No restrictions on publication year were applied, but only articles published in English were considered. Studies were assessed for population overlap according to geographical location, timeframe, sample size, outcome under study, and statistical methods. In case of overlap, the most recent or largest for its biomarker was selected.

Data were extracted using a standardized spreadsheet including publication details (authors, year, journal), study characteristics (geographical region, design, population, sample size), sample demographics (age, sex, smoking, body mass index, hypertension, diabetes, CV disease, cerebral disease), AIx measurement (method, definition, quantification scale), cSVD neuroimaging markers (marker, imaging modality, definition, quantification scale), and statistical analysis information (type, effect estimates, 95% confidence intervals [CIs], and adjusting variables). Corresponding authors were contacted when relevant data were described but not quantified to request additional analyses. Disagreements between reviewers were solved through team consensus.

### PWRs biomarkers and outcomes

The exposure variables of interest included PWRs biomarkers, expressed as continuous variables: AIx and AIx corrected for heart rate of 75 bpm (AIx75). Further details are provided in the **Supplementary Material**. The primary outcomes were neuroimaging markers of cSVD defined according to STandards for ReportIng Vascular changes on nEuroimaging (STRIVE)^28^: lacunes, WMH, CMB, and PVS. Studies assessing lacunes and WMH using either magnetic resonance imaging (MRI) or computed tomography (CT) were considered eligible, as prior research supports the validity of both methods.^32,33^ However, only MRI-based studies were included for evaluating CMB and PVS. Additional details on biomarkers and definitions applied for cSVD are described in the **Supplementary Material**.

### Risk of bias assessment

The risk of bias in the included studies was assessed using the Newcastle-Ottawa scale.^34^ Given that most eligible studies were of cross-sectional or cohort design, the nine-item cohort subscale was applied uniformly. The evaluation criteria included representativeness of the exposed population, selection of the non-exposed group, ascertainment of exposure (AIx), absence of the outcome (cSVD) at study onset, comparability of the exposed and non-exposed groups based on age and hypertension, and quality of outcome assessment. Cross-sectional studies, by definition, received no points for longitudinal assessment items (outcome absence at baseline, follow-up period length, and completion). Detailed, predefined criteria for each item specific to this review are provided in the **Supplementary Table S1**.

### Statistical analysis

For each eligible study, estimates and 95% CIs of associations between PWRs biomarkers and cSVD neuroimaging markers were extracted. Data for the association between PWRs with cSVD (9 studies) were harmonized to standardized beta coefficients to enable meta-analysis. Unstandardized coefficients were standardized using the standard deviation (SD) of PWR and cSVD; when SDs were not reported, they were estimated from available data following established methods.^35^ Most studies provided correlation coefficients (OR), which were transformed into standardized beta coefficients (β) using the formula β=ln(OR), as described by Szumilas.^36,37^ All other conversions were performed using validated formulas via the online effect size calculator tool (https://www.campbellcollaboration.org/calculator/).^38^

Random-effects meta-analyses of standardized beta coefficients were conducted using the DerSimonian and Laird method^39^ as the primary approach. Separate analyses were performed for each association between PWRs biomarkers and cSVD markers. Between-study heterogeneity was assessed using the I^2^ statistic, with significance evaluated through the Cochran Q test. Subgroup analyses were conducted to investigate heterogeneity sources, stratified by levels of adjustment for confounding factors, including age, sex, BP, hypertension, and heart rate. Statistical significance for the primary analyses was set at a two-sided *P* value of < 0.05. All statistical analyses were performed using STATA Software, version 13.0 (Stata Corporation, College Station, TX).

## RESULTS

### Systematic literature review

The study selection process is summarized in the flowchart diagram (**Supplementary Figure S1**). An initial screening of 1,129 articles from the literature identified 36 studies that met the eligibility criteria. After further review, 10 articles remained for potential inclusion. Despite contacting the corresponding author for missing data on the association between AIx and WMH, the necessary information was not provided, leading to the study’s exclusion (see **Supplementary Table S2** for detailed exclusion reasons). Consequently, a total of nine studies, comprising 6,774 participants, were included in the final meta-analysis.

### Studies characteristics per type of PWRs and cSVD biomarker

Out of the nine identified studies on AIx (**Tables 1–3**), all,^20,40–47^ encompassing a total of 6,774 participants, measured AIx at central arteries (carotid artery or aorta). None of the studies assessed AIx at peripheral arteries (radial artery or finger). Aortic AIx was evaluated in five studies,^41,42,45–47^ involving 5,297 participants, using radial applanation tonometry and PWA with generalized transfer functions (GTFs) (SphygmoCor, ATCOR Medical, Sydney, Australia), in all but one study,^45^ which utilized brachial oscillometry with PWA and GTF (Skidmore Medical Vicorder device). Carotid AIx was analyzed in five studies,^20,40,43,44,47^ comprising 1,763 participants, using local carotid pulse wave recordings. Applanation tonometry was used in three studies (*n* = 1,159), with three different systems,^20,43,47^ while ultrasonography was employed in three studies (*n* = 890) with three different systems.^40,44,47^ Two of these studies,^40,47^ with 786 participants, assessed carotid pulse waveforms using carotid flow measurements. No studies examining PWR biomarkers other than Aix were found in relation to cSVD. No studies provided data for the association between AIx75 and cSVD. Further details on study design, population characteristics, and PWR measurement methodologies are provided in **Table 1** and **Table 2**.

**Table 1.** Characteristics of studies investigating the association between augmentation index (AIx, %) and cerebral small vessel disease (cSVD)..

Study	Study design	Population type	Region	No.	PWRSs: Device/method/arterial site recording	PWRs site estimation
Ochi N. et al (2010)	Cross-sectional	Middle aged to elderly persons	Japan	500	Aloka, SSD-3500SV/Flow AIx measured according to Hirata et al.^a^ / carotid	^b^Carotid
Kearney-Schwartz A. et al (2009)	Cross-sectional	High CVD: elderly hypertensive patients	France	184	SphygmoCor / applanation tonometry & PWA & GTF / radial	Aorta
Inkeri J. et al (2021)	Cohort	High CVD: diabetes mellitus type 1	Finland	186	SphygmoCor / applanation tonometry & PWA & GTF / Radial	Aorta
Nakano T. et al (2012)	Cross-sectional	General population	Japan	205	Form PWV/ABI / Tonometry^c^ & PWA / carotid	Carotid
Turk M. et al (2015)	Case-control	Selected population: patients with ischemic leukoaraiosis	Slovenia	104	Alpha 10 / ultrasound^c^ & PWA / carotid	Carotid
Mitchell G. et al (2011)	Cohort	Older (69–93 years of age), community-based sample	Iceland	668	custom transducer / arterial tonometry (carotid pressure waveform)/ carotid	Carotid
van Hout M. et al (2021)	Cohort	General randomly selected population (middle-aged)	United Kingdom	4207	Skidmore Vicorder / brachial sphygmomanometer & GTF & PWA / brachial	Aorta
Gutierrez J. et al (2019)	Cohort	General randomly selected population	United States	434	SphygmoCor / applanation tonometry & PWA & GTF / radial	Aorta
Hashimoto J. et al (2018)	Cross-sectional	High CVD risk: hypertension	Japan	286	SphygmoCor / applanation tonometry & PWA & GTF / radial AND Vivid i, ultrasound / ultrasound^d^ / carotid	Aorta and ^b^carotid

**Abbreviations:** AIx, augmentation index; AP, augmentation pressure; CVD, coronary vascular disease; cSVD, cerebral small vessel disease; GTF, generalized transfer functions; PP, pulse pressure; PWA, pulse wave analysis; PWR, pressure wave reflection; PWV, pulse wave velocity; V1, early systolic peak (or shoulder) velocity; V1h, early systolic velocity wave height; V2, late systolic peak (or shoulder) velocity; V2h, late systolic velocity wave height; Vd, end-diastolic velocity; Ved, end-diastolic flow velocity; Vs, peak systolic flow velocity; Vsr, peak flow velocity of the secondary rise in the common carotid flow velocity waveform; Vmax, systolic maximum velocity.

^a^Flow AIx measured according to Hirata et al., Flow AI was defined as (Vsr − Ved)/(Vs − Ved).

^b^The study provides data for carotid flow AIx.

^c^AIx = AP/PP %.

^d^AIx was calculated with the equations: FAIx = V2h ÷ V1h = (V2-Vd) ÷ (V1-Vd) [%] and FAIx2 = (V2-V1) ÷ (Vmax-Vd) [%].

*Device’s details:*

Aloka, SSD-3500SV, Tokyo, Japan .

SphygmoCor PWV Medical Pty Ltd, Ermington, Sidney, Australia.

SphygmoCor, ATCOR Medical, Sydney, NSW, Australia.

Form PWV/ABI, Colin Medical Technology, Komaki, Japan.

Alpha 10, Aloka, Tokyo, Japan.

Custom transducer (Cardiovascular Engineering, Inc.).

Vicorder (Skidmore Medical) device.

Vivid i, GE Healthcare, Tokyo, Japan.

**Table 2.** Characteristics of studies investigating the association between augmentation index (AIx, %) and cerebral small vessel disease (cSVD).

Study	AIx (%, mean)	AIx SD	Age (years, mean)	Male sex (%)	BMI (mean)	HTN (%)	DM (%)	DLD (%)	CAD (%)	Stroke (%)	Sm (%)
Ochi N. et al (2010)	Carotid flow AIx SCI(+): 72 SCI(−): 70.2	SCI(+):10.8 SCI(−): 13.3	66.9	38	23.1	57	11	66	0	10.4	6
Kearney-Schwartz A. et al (2009)	37.7	9.8	69.3	48	27.8	100	12	#	#	0	39 (current and past)
Inkeri J. et al (2021)	14.9	6.6	40 (median)	47.8	26.2	#	100	#	#	0	#
Nakano T. et al (2012)	21	14	57	54	23.7	#	#	#	#	0	#
Turk M. et al (2015)	ILA:18.78 C:16.61	ILA: 11.49 C:8.74	ILA:54.9 C:53.2	57	ILA:30.2 C:29.6	ILA: 68 C: 56	0	ILA: 66 C:73	0	0	ILA: 20 C: 18
Mitchell G. et al (2011)	Fm:17 M:12	Fm:17 M:13	Fm:75 M:76	43	Fm:27 M:27	#	Fm:8 M:13	#	Fm:7 M:30	0	Fm:12 M:11
van Hout M. et al (2021)	21.2 M = 19.4 Fm = 22.7	8.6 M = 8 Fm = 8.8	61.1 M = 61.7 Fm = 60.5	46	26.4 M = 26.8 Fm = 26	23.5 M = 27.4 Fm = 20.1	4.4 M = 5.5 Fm = 3.4	#	3.4 M = 5.2 Fm = 1.9	1.2 M = 1.7 Fm = 0.7	4.5
Gutierrez J. et al (2019)	#	#	71	40	#	85	24	68	19	0	16
Hashimoto J. et al (2018)	Aorta = 27 carotid pressure = 21 carotid flow = 65	Aorta = 12 carotid pressure = 19 carotid flow = 26	54	40	24.8	100	26	43	#	0	#

**Abbreviations:** AIx, augmentation index; BMI, body mass index; C, controls; CAD, coronary artery disease; cSVD, cerebral small vessel disease; DM, diabetes mellitus; DLD, dyslipidemia; Fm, female; HTN, hypertension; ILA, ischemic leukoaraiosis; M, male; PWR, pressure wave reflection; SCI, silent cerebral lacunar infarcts; SD, standard deviation; SM, smoking.

^#^Data not mentioned

**Table 3.** Characteristics of studies investigating the association between augmentation index (AIx, %) and cerebral small vessel disease (cSVD).

Study	Imaging method / magnetic field (Tesla )	Method of quantification/scale used for assessment of SVD	cSVD assessed
Ochi N. et al (2010)	MRI/3.0 T	Qualitative/NA	Lacunes
Kearney-Schwartz A. et al (2009)	MRI/1.5 T	Semi-quantitative/Fazekas and Schmidt	WMH
Inkeri J. et al (2021)	MRI/3.0 T	Semi-quantitative/Fazekas, STRIVE criteria	WMH, CMB
Nakano T. et al (2012)	MRI/NA	Semi-quantitative/classification PICA	WMH
Turk M. et al (2015)	MRI/1.5 T	Semi-quantitative/Fazekas	WMH
Mitchell G. et al (2011)	MRI/1.5 T	Quantitative/NA	WMH
van Hout M. et al (2021)	MRI/3.0 T	Quantitative/BIANCA	WMH
Gutierrez J. et al (2019)	MRI/1.5 T	Quantitative/NA	WMH
Hashimoto J. et al (2018)	MRI/1-1.5 T	Semi-quantitative/Fazekas	WMH

**Abbreviations:** AIx: augmentation index; BIANCA: brain intensity abnormality classification algorithm; CMB: cerebral microbleeds; cSVD: cerebral small vessel disease; MRI: magnetic resonance imaging; NA: not applicable; STRIVE: standards for reporting vascular changes on neuroimaging; T: tesla; WMH: white matter hyperintensities

Of the nine studies assessing cSVD, one study examined Aix in relation to lacunes,^40^ one investigated Aix and CMB,^41^ and eight focused on WMH.^20,41–47^ All but two studies treated cSVD as a qualitative variable. The two studies^20,46^ that evaluated WMH as a continuous variable were analyzed separately, which did not alter the overall association between AIx and cSVD. MRI was used for cSVD assessment in all studies, with no data reported from CT imaging. No eligible studies investigating PVS were identified. Methodological details for each study are summarized in **Table 3**.

### Meta-analysis on AIx and cSVD

**Figure 1.**
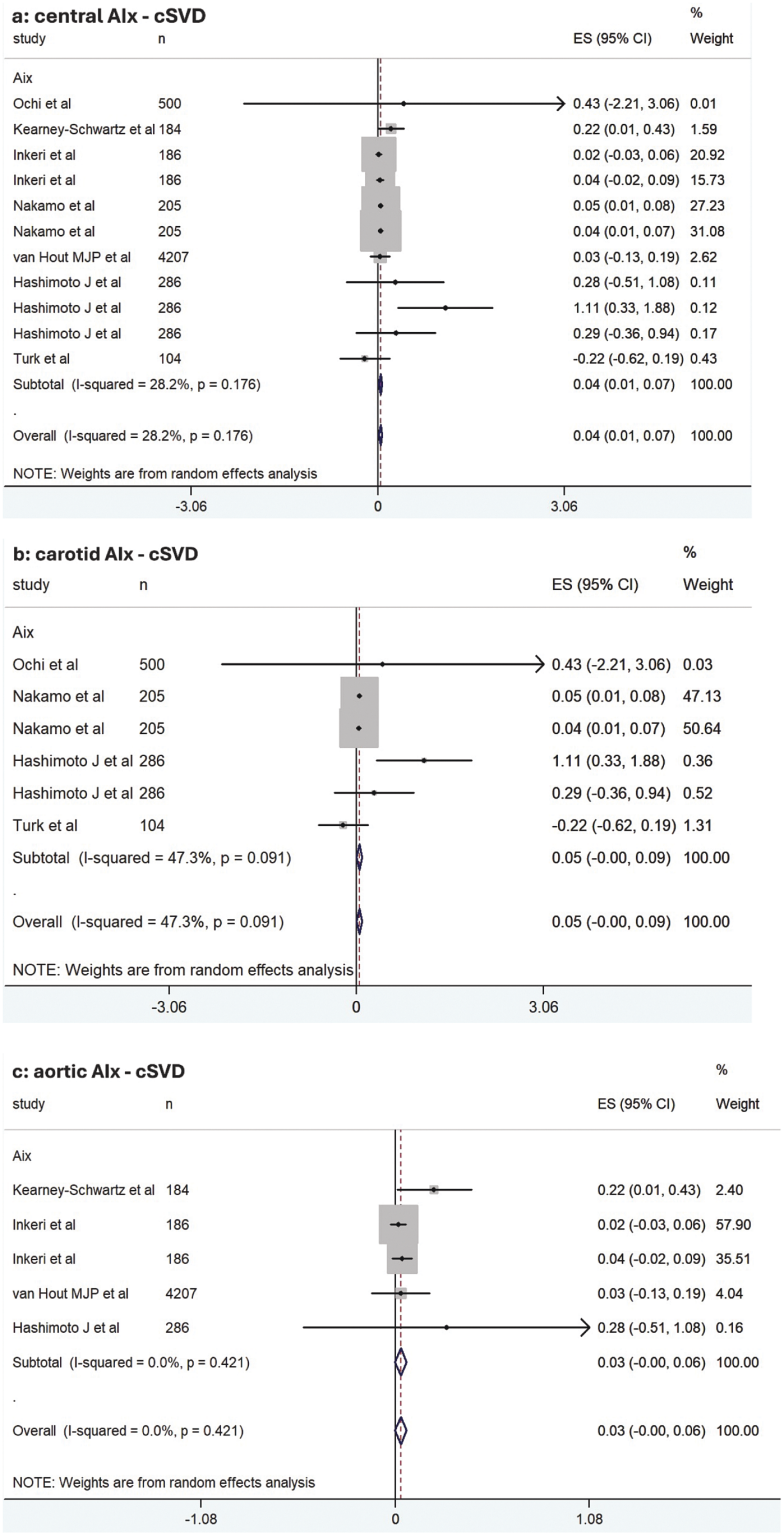
Forest plot of the meta-analysis association estimates between: **(a)** central (aortic or carotid) augmentation index (AIx), **(b)** carotid AIx, **(c)** aortic AIx, and cerebral small vessel disease (cSVD) in all eligible studies.

Meta-analysis was performed separately for AIx measured at central arteries (carotid artery or aorta), carotid artery, and aorta. Central AIx (carotid or aortic) demonstrated a marginal association with cSVD (standardized beta coefficient [β]: 0.04, 95% CI: 0.01 to 0.07) (**Figure 1a**).^40–45,47^ When studies using models adjusted for at least age, sex, and BP were included, a similar marginal association was observed (β: 0.04, 95% CI: 0.02 to 0.07) (**Supplementary Figure S2a**).^40–43,45,47^ However, no significant association was found when models were further adjusted for heart rate (β: 0.33, 95% CI: −0.11 to 0.78) (**Supplementary Figure S2b**).^45,47^ Excluding studies using flow-based AIx measurements yielded a marginal association (β: 0.04, 95% CI: 0.02 to 0.06) (**Supplementary Figure S2c**).^41–45,47^

Carotid Aix, analyzed separately from aortic Aix, was not significantly associated with cSVD (β: 0.05, 95% CI: 0.00 to 0.09) (**Figure 1b**).^40,43,44,47^ Including studies with models adjusted for age, sex, and BP yielded the same result (β: 0.05, 95% CI: 0.00 to 0.09) (**Supplementary Figure S3a**).^40,43,47^ However, a significant association was observed when flow-based Aix measurement studies were included (β: 1.05, 95% CI: 0.30 to 1.80) (**Supplementary Figure S3b**).^40,47^

Aortic Aix, separately analyzed, showed no significant association with cSVD (β: 0.03, 95% CI: 0.00 to 0.06) (**Figure 1c**).^41,42,45,47^ Including studies with models adjusted for age, sex, BP, and heart rate did not change this finding (β: 0.04, 95% CI: −0.12 to 0.20) (**Supplementary Figure S4a**).^45,47^

### Meta-analysis on Aix and WMH

**Figure 2.**
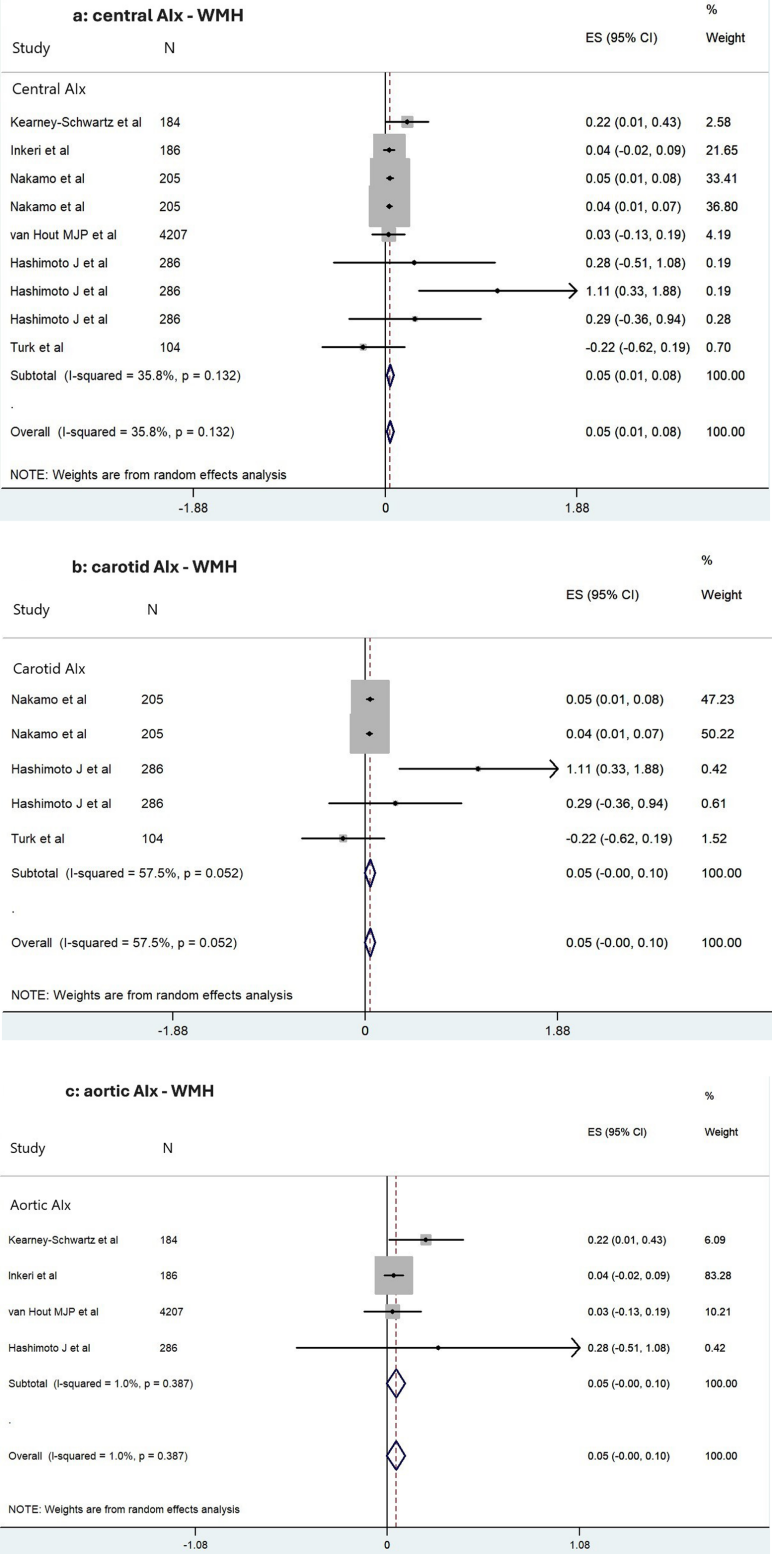
Forest plot of the meta-analysis association estimates between: **(a)** central (aortic or carotid) augmentation index (AIx), **(b)** carotid AIx, **(c)** aortic AIx, and white matter hyperintensities (WMH) in all eligible studies. In all figures, standardized beta coefficients (β) of each study are depicted as data markers; shaded boxes around the data markers indicate the statistical weight of the respective study; 95% confidence intervals (CI) are indicated by the error bars; pooled effect estimates along with their 95% CI are reflected as a diamond. All the analyses were conducted with the STATA Software version 13.0 (Stata Corporation, College Station, TX). Abbreviations for all figures: N, number of individuals; ES, effect size; CI, confidence intervals.

Central AIx (carotid or aortic) showed a marginal association with WMH (β: 0.05, 95% CI: 0.01 to 0.08) (**Figure 2a**).^41–45,47^ A similar marginal association was observed when studies using flow AIx were excluded (β: 0.04, 95% CI: 0.03 to 0.06) (**Supplementary Figure S5a**).^41–45,47^ Carotid Aix, analyzed separately from aortic Aix, was not significantly associated with WMH (β: 0.05, 95% CI: 0.00 to 0.10) (**Figure 2b**).^43,44,47^ However, excluding flow AIx measurements revealed a marginal positive association (β: 0.04, 95% CI: 0.02 to 0.06) (**Supplementary Figure S5b**).^43,44,47^ Aortic Aix, analyzed independently from carotid Aix, demonstrated no significant association with WMH (β: 0.05, 95% CI: 0.00 to 0.10) (**Figure 2c**).^41,42,45,47^

### Risk of bias assessment

The overall quality of the included studies was moderate. One study^20^ satisfied nearly all criteria of the Newcastle-Ottawa scale, scoring seven out of nine, with a median score of 6 across all studies. Nearly all studies met the criteria for appropriate outcome assessment methods. High-quality or acceptable PWA methodologies were employed in four studies (44.4%).^20,45–47^ None of the studies included longitudinal follow-up of participants. The association between AIx and WMH was analyzed in eight studies (88.9%) with a median quality score of 6/9.^20,41–47^ A single study (11.1%) investigated the association between AIx and lacunes, achieving a quality score of 6/9,^40^ while another study (11.1%) examined AIx and CMBs, also scoring 6/9.^41^ Five studies (55.6%) assessed aortic AIx and cSVD with a median quality score of 6/9^41,42,45–47^ and five studies (55.6%) analyzed carotid AIx and cSVD, also with a median score of 6/9.^20,40,43,44,47^ General population-based studies accounted for 33.3% (three of nine) with a median score of 6/9,^43,45,46^ while studies focused on high CVD risk populations comprised 44.4% (4 of 9) with the same median quality score.^41,42,44,47^ Fully adjusted models (age, sex, BP, and heart rate) were used in four studies (44.4%) with a median score of 6/9.^20,45–47^ Models adjusted for age, sex, and BP were utilized in three studies (33.3%) with a median quality score of 5/9,^40–43^ and one study (11.1%) employed unadjusted models, scoring 3/9^44^ (**Supplementary Table S3**).

## DISCUSSION

Higher AIx measured at central arteries (carotid and aorta) showed a marginal positive association with cSVD and WMH; however, these associations are likely to have limited clinical relevance. The lack of a clinically meaningful link between AIx and cSVD warrants careful consideration.

First, AIx may not be the most suitable metric for quantifying PWRs. It is derived solely from pressure waveform analysis and represents a composite measure influenced by multiple parameters, including heart rate, wave reflections amplitude, peripheral resistance, and arterial stiffness.^48^ Additionally, mechanistic simulations using computational models by Heusinkveld et al. demonstrated that AIx is significantly affected by myocardial shortening velocity.^49^

Second, although AIx effectively reflects the adverse impact of elevated PWRs on the left ventricle,^3^ regardless of the arterial site of assessment, it correlates more weakly with LVH compared to P_b_.^3^ P_b_, introduced by Westerhof et al.,^2^ is derived using the impedance method, assuming a steady-state CV condition, requiring simultaneous pressure and flow measurements at a single arterial location. Unlike AIx, P_b_ does not rely on identifying the inflection point between forward and reflected waves, making it a more robust measure of PWRs. Future studies examining P_b_ in relation to cSVD may offer improved insights.

Third, disproportionate increases in aortic characteristic impedance as compared with carotid input impedance, as observed in elderly or early vascular aging,^20^ may reduce proximal wave reflection, allowing heightened pulsatility transmission to the brain microcirculation, with detrimental effects.^47,50^ This suggests that maintaining a balance of PWRs or generating localized reflections at the micro-macrocirculation interface, possibly due to arteriolar remodeling, could serve as an adaptive mechanism protecting against pulsatile stress. An inverse relationship between locally generated PWRs and cSVD could be hypothesized, but demonstrating this requires sophisticated methodologies for direct local PWR and reflection coefficient assessments, rarely performed in existing studies,^20^ though with supportive findings.^20^

The present meta-analysis, focusing on centrally measured Aix, captures global rather than localized wave reflection phenomena. Therefore, the modest association between AIx and cSVD may reflect the composite nature of Aix, which merges information about the overall status of the circulation (heart, total peripheral resistance, global PWRs), without isolating local effects. This explains why the present meta-analysis’ findings contrast with the results of a previous recent meta-analysis that investigated the relationship between PWRs και LVMi, since the latter assessed PWRs and organ damage at the same level.^3^

Several limitations of this meta-analysis must be acknowledged. First, although wave reflections theory has significantly enhanced the understanding of heart-arterial interactions,^2^ important uncertainties remain, including the precise locations of major reflections sites.^5^ Moreover, the present meta-analysis was conducted using study-level data rather than individual participant data, which limits the granularity and precision of analyses. The heterogeneity across studies was notable, encompassing differences in population characteristics, outcome definitions, and the technologies used for measuring PWRs and cSVD, as well as variations in adherence to STRIVE criteria for cSVD biomarkers. These methodological inconsistencies hinder the comparability of findings and introduce variability into the pooled estimates. In addition, there were unfortunately no studies that provided data for the association between AIx75 and cSVD; whether this would substantially modify the findings, taking into account the strong dependency of AIx on heart rate, will remain unknown. Yet, at least some of the studies^20,45–47^ provided results adjusted for heart rate, therefore the strong dependency of PWRs on heart rate is at least partly taken into account.

Furthermore, this meta-analysis was limited by the age range of participants in the included studies, with mean ages spanning from 40 to 70 years but mainly representing ages older than 60 years. This age restriction may have influenced the generalizability of the findings, as AIx, as a measure of arterial wave reflection, is known to vary with age and given the rather narrow age range, the relatively small effects are not entirely unexpected. Central AIx is considered a more sensitive marker of arterial aging in younger individuals, whereas its predictive value may differ in older populations.^51^ The dynamics of arterial wave reflection, including AIx, change throughout the lifespan, potentially influencing its association with cSVD. Additionally, the pathophysiology of cSVD evolves with age,^52^ suggesting that the mechanisms linking AIx to cSVD may differ between younger and older populations. Future research incorporating age-stratified analyses is essential to better understand the age-dependent effects of AIx on cSVD and improve the clinical applicability of these findings. Lastly, publication bias and limited control over data handling further constrain the generalizability of results. Since the included studies vary in design quality and population scope, caution is warranted when applying these findings to broader populations. Future investigations should prioritize individual participant data analyses and standardized measurement methodologies to enhance the robustness and applicability of evidence on PWRs and cSVD relationships.

## PERSPECTIVES

The evidence on the potential role of PWRs in the development is currently under investigation and expanding in other neurodegenerative fields and diseases such as Alzheimer’s disease.^30^ While the present data do not establish a robust and clinically meaningful association between AIx and cSVD, they highlight the need for further research. Future studies should focus on advancing methodologies capable of assessing local PWRs and reflection coefficients. Such approaches may provide deeper insights into the relationship between PWRs and brain microcirculation, enhancing our understanding of their potential role in the pathophysiology of cSVD and related neurodegenerative conditions.

## Supplementary material

Supplementary materials are available at *American Journal of Hypertension* (http://ajh.oxfordjournals.org).

hpaf054_suppl_Supplementary_Tables_S1-S3_Figures_S1-S51

## Data Availability

The data that support the findings of this study are available from the corresponding author upon reasonable request.
