# Supplementary material for: Association of Augmentation Index With Cerebral Small Vessel Disease: A Systematic Review and Meta-Analysis
Source: Am J Hypertens. 2025 Apr 19;38(9):686–96. doi: 10.1093/ajh/hpaf054 (PMC12354286; doi:10.1093/ajh/hpaf054)

**Association of augmentation index with cerebral small vessel disease: A systematic review and metanalysis**

**Supplementary methods:**

*A. Search algorithm*

Our literature search was performed on the PubMed database (<https://www.ncbi.nlm.nih.gov/pubmed/>) using the following combination of search terms (algorithm):

(("wave reflections" OR "pressure wave reflections" OR "augmentation index" OR AIx OR "backward pressure wave" OR Pb OR "backward wave" OR "amplitude of backward wave" OR "reflection pressure" OR RP OR "reflection index" OR RI OR "reflection magnitude" OR RM)) AND ((((cerebral OR brain) AND (microangiopathy OR micro-angiopathy OR microvessel OR "small vessel" OR small-vessel OR microvascular OR microbleed OR microhemorrhage OR dot-like hemosiderin OR leukoaraiosis OR "Virchow-Robin" OR (perivascular AND space) OR ((lacunar OR lacunae OR lacunes) AND (infarct OR stroke)))) OR ("white matter" AND (disease OR diseases OR hyperintensity OR lesion OR lesions))))

The final search was performed on 31 December 2023 and yielded 1129 articles. Thus, 966 articles derived through PubMed and 163 articles derived through Scopus, were cumulatively screened for eligibility. Articles derived through the search were sorted by publication date.

B. *Pressure wave reflections: biomarkers and definitions applied*

**Augmentation index (AIx)**: describes the relative enhancement of central blood pressure by pressure wave reflections and is defined by the formulas: AIx=100 x AP/PP, where PP is the aortic pulse pressure (systolic minus diastolic pressure) or AIx=P2/P1, where P2 is the second (late) systolic peak and P1 the first systolic peak [1,6,7].

**Heart-rate corrected wave reflections (AIx75)**: describes the augmentation index adjusted at heart rate 75 bpm [1,6,7].

*C. Cerebral small vessel disease: biomarkers and definitions applied*

The primary outcomes of our study included the following neuroimaging markers of cSVD, according to STandards for ReportIng Vascular changes on nEuroimaging (STRIVE) [28]: lacunes, white matter hyperintensities (WMH), cerebral microbleeds (CMB), and perivascular spaces (PVS). For the assessment of lacunes and WMH eligible were considered studies using either Magnetic Resonance Imaging (MRI) or Computed Tomography (CT) as previous literature has described compliance validity between the two methods [32,33]. For the evaluation of CMB and PVS only studies using MRI have been considered.

We included studies defining lacunes as round or ovoid, subcortical, fluid-filled cavities, measuring between 3 and 15-mm in maximal diameter, consistent with a previous acute small deep brain infarct or haemorrhage in the territory of one perforating arteriole. WMH have been identified as hyperintense areas on T2-weighted MRI sequences, isointense or hypointense on fluid-attenuated inversion recovery (FLAIR) imaging or as CT hypodensities. Only studies assessing WMH presence or severity through semi-quantitative visual rating scales (e.g., Fazekas) or WMH volume via automated or semi-automated methods have been considered. CMB had to be visualized as small (≤10 mm) areas of signal void with associated blooming on T2-weighted MRI sequences. PVS have been defined as fluid-filled spaces following the course of a vessel with cerebrospinal fluid-like signal intensity, visualized in MRI.

**Supplementary Figures & Tables**

**Table S1.** Management of the quality scoring criteria of the cohort subscale of the Newcastle-Ottawa assessment scale for the purposes of the current study.

|  | **Selection** | | | | **Comparability** | **Outcome** |
| --- | --- | --- | --- | --- | --- | --- |
| **Criterion**  **Scoring** | ***Representativeness of the exposed cohort*** | ***Selection of the non-exposed cohort*** | ***Ascertainment of exposure*** | ***Exclusion of outcome presence at start of study*** | ***Comparability for Age, Sex, Heart Rate and Blood Pressure*** | ***Assessment of outcome - Quality of measurement*** |
| Point  awarded if… | General population-based study | Drawn from the same community as the exposed cohort | Detailed report about the quality of Pulse Wave Analyses Methodology  (fasting hours before examination, report of medication/abstain from vasoactive medication or other substances the day of examination, body posture at the time of medication, time of rest before measurement, description of device and software used, description of procedure for PWA assessment, description of site of PWR acquisition, number of measurements performed, assessment of waveforms quality) | Cohort studies in which the outcome addressed is assessed at baseline and either subjects with the outcome are excluded from the study or the increment of the outcome is assessed | **1.** Two points awarded if: Adjustment for Age, Sex, HR and BP  **2.** One point awarded if: adjustment for Age, Sex and HR  **3.** One point awarded if: Adjustment for Age, Sex and +/- BP | **1.** MRI quality (>=3Tesla)  **2.** Clearly defined MRI protocol  **3.** Standardized definition of outcome  **4.** >1 cSVD markers assessed  **5**. standardized rating scale  **6.** blinded assessment of MRI findings  (one point awarded for every two quality indicators fulfilled) |
| Point **not** awarded if… | **1.** High-risk population-based study (hypertensive, patients with cardiovascular history etc.)  **2.** Random sample | **1.** Drawn from a different source  **2.** No description of the derivation of the non-exposed cohort | **1.** Poor quality  **2.** Method used not reported | **1.** Cross-sectional studies,  **2.** Cohort studies which do not fulfil the above criteria | Unadjusted studies for these parameters | **1.** Method not reported  **2.** Studies which does not fulfil the above criteria |

Abbreviations: BSA: body surface area; BMI: body mass index; HR: heart rate; BP: blood pressure, PWA: pulse wave analyses; PWR: pressure wave reflection; MRI: magnetic resonance imaging; cSVD: cerebral small vessel disease

**Table S2:** Number of articles excluded after screening the full-text by aetiology of exclusion.

| **Aetiology of exclusion** | **Number of articles** |
| --- | --- |
| Lack of direct correlation between pressure wave reflection biomarkers and cSVD. | 12 |
| Articles not presenting relevant quantitative data - author contacted but did not reply | 1 |
| Study population not eligible (rare disorders, genetic diseases predisposing to cerebral small vessel structural changes e.g. rare genetical disorders etc.) | 2 |
| Sample size less than 100 participants | 3 |
| Underage sample article (<18yrs) | 1 |
| Exposure or outcome assessed was not relevant | 5 |
| In vitro and animal-based papers | 1 |
| Reviews, systematic reviews, narrative reviews, meta-analyses, case reports, case series | 2 |
| **Total articles excluded** | **27** |

Abbreviations: cSVD: cerebral small vessel disease

**Table S3:** Results of the quality assessment of eligible studies according to the cohort subscale of the Newcastle-Ottawa scale. Articles are sorted in order of decreasing total quality score.

| Author | Selection | Comparability | Outcome | Total Score |
| --- | --- | --- | --- | --- |
| Mitchell et al | ☆★★☆ | ★★ | ★★★ | 7 |
| Ochi et al | ★★★☆ | ★☆ | ★★☆ | 6 |
| Inkeri et al. | ☆★★☆ | ★☆ | ★★★ | 6 |
| van Hout MJP et al | ★★☆☆ | ★★ | ★★☆ | 6 |
| Gutierrez J et al | ★★☆☆ | ★★ | ★★☆ | 6 |
| Hashimoto J et al | ☆★★☆ | ★★ | ★★☆ | 6 |
| Kearney-Schwartz et al. | ☆★☆☆ | ★☆ | ★★☆ | 4 |
| Nakamo et al | ★★☆☆ | ☆☆ | ★☆☆ | 3 |
| Turk et al | ☆★☆☆ | ☆☆ | ★★☆ | 3 |

*Selection items include:* representativeness of the exposed cohort, selection of the non-exposed cohort, ascertainment of exposure, and exclusion of outcome presence at start of study.

*Comparability items include***:** comparability for age, sex, heart rate and blood pressure.

*Outcome items include:* assessment of outcome.

**Figure S1**. Flowchart of the study selection process.


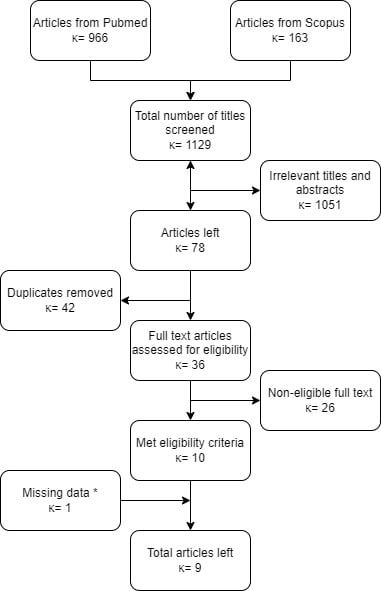


*Missing (after contacting with authors) or unusable data for meta-analyses.

**Figure S2.** Forest plot of the meta-analysis association estimates between central augmentation index (AIx) and cerebral small vessel disease (cSVD) in:

**(S2a) studies that used** adjusted models (at least for age, blood pressure and sex),

**(S2b) studies that used** adjusted models (at least for age, blood pressure, sex and heart rate),

**(S2c) all studies after excluding** flow Aix,

In all figures, standardized beta coefficients (β) of each study are depicted as data markers; shaded boxes around the data markers indicate the statistical weight of the respective study; 95% confidence intervals (CI) are indicated by the error bars; pooled-effect estimates along with their 95% CI are reflected as a diamond. All the analyses were conducted with the STATA Software version 13.0 (Stata Corporation, College Station, TX, USA).

Abbreviations for all figures: N: number of individuals; ES: effect size; CI: confidence intervals

**Figure S2a**: central Aix (adjusted for age, sex, BP) – cSVD


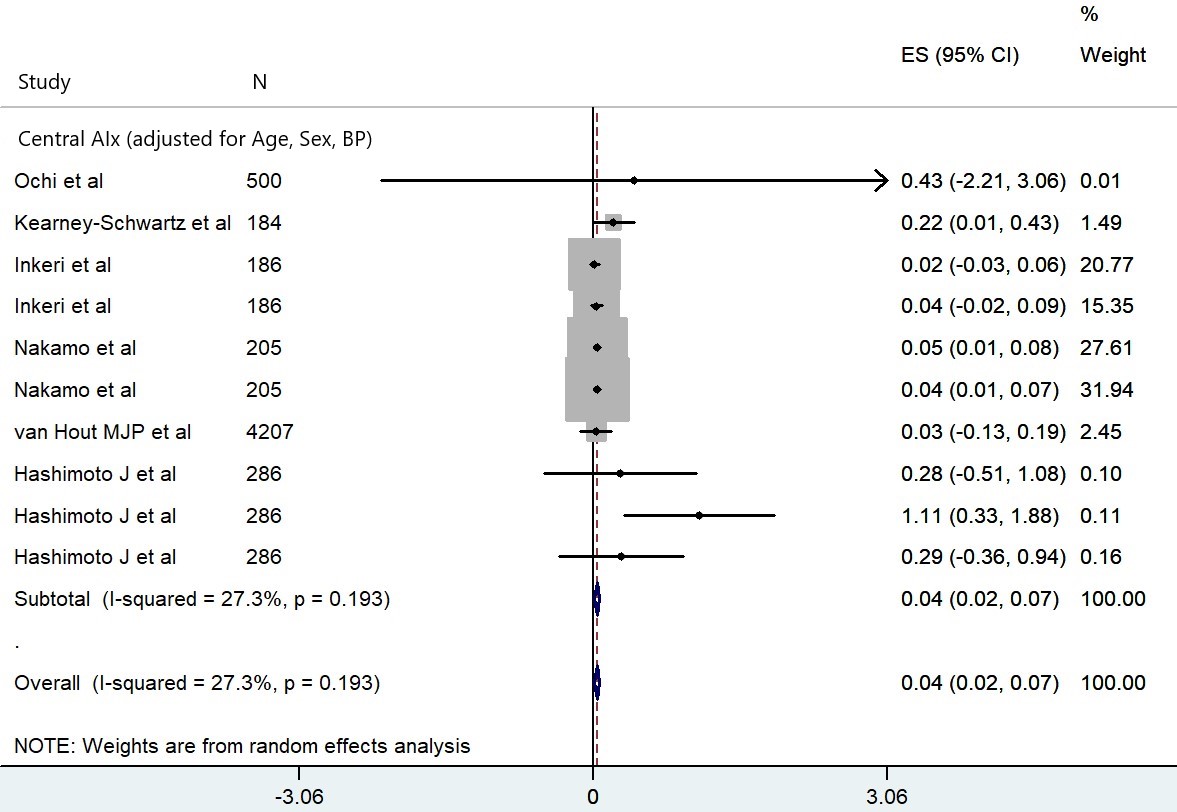


**Figure S2b**: central Aix (adjusted for age, sex, BP, HR) – cSVD

**
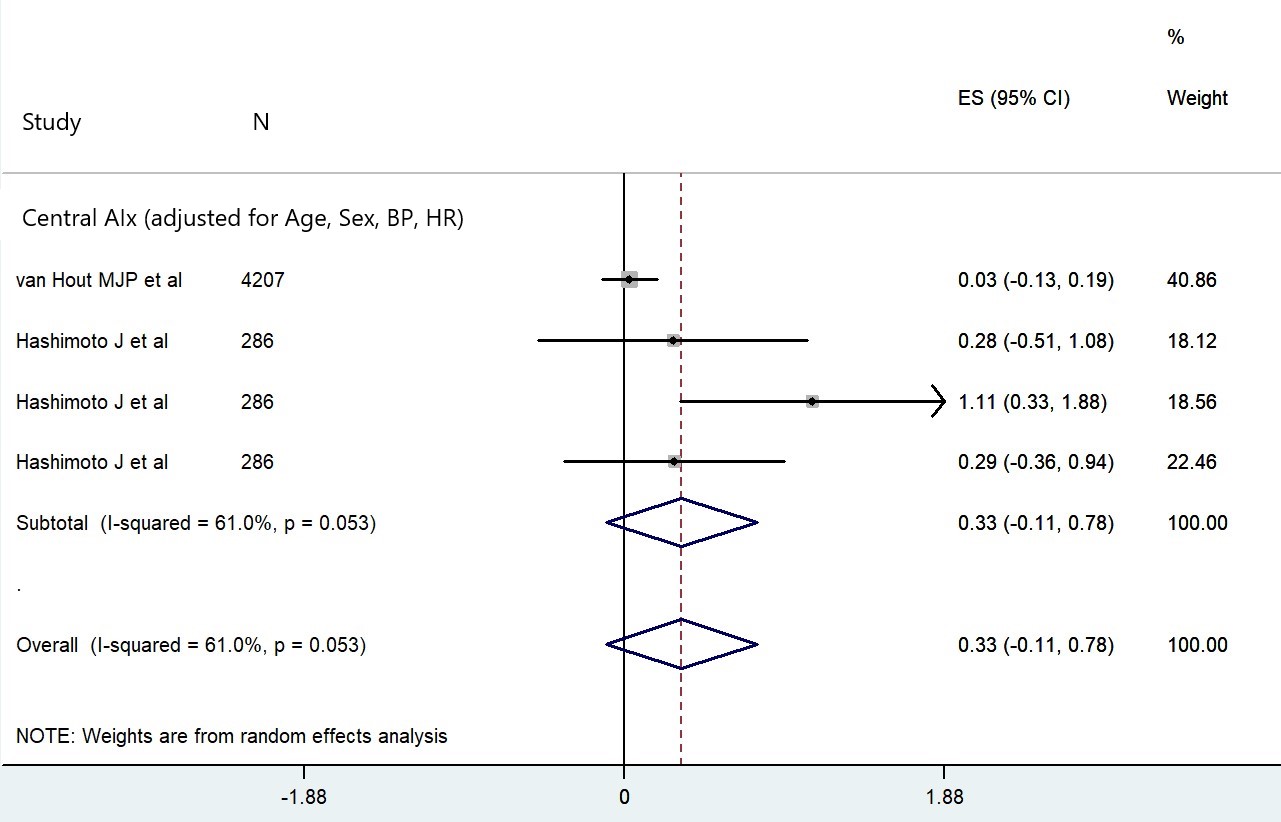
**

**Figure S2c**: central Aix (**without** flow Aix studies) – cSVD


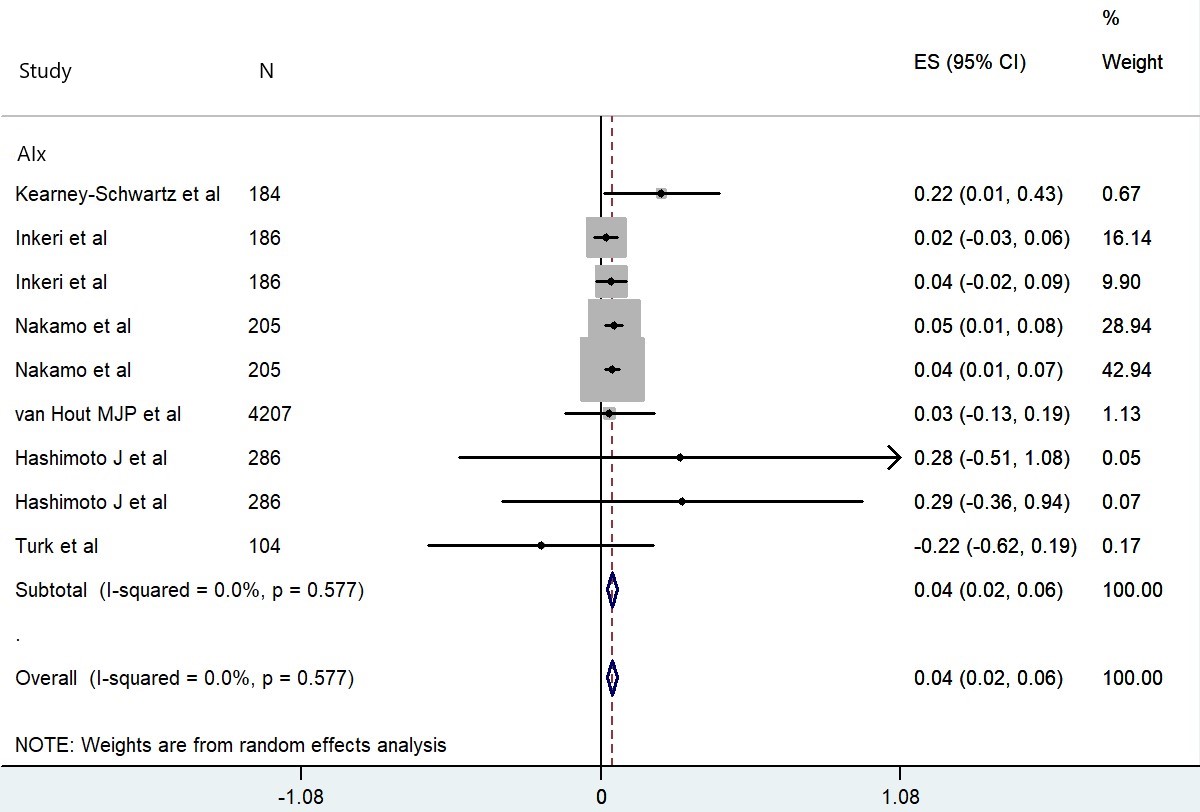


**Figure S3.** Forest plot of the meta-analysis association estimates between carotid AIx and cSVD in:

**(S3a) studies that used** adjusted models (at least for age, blood pressure and sex),

**(S3b) all studies that used** flow Aix,

In all figures, standardized beta coefficients (β) of each study are depicted as data markers; shaded boxes around the data markers indicate the statistical weight of the respective study; 95% confidence intervals (CI) are indicated by the error bars; pooled-effect estimates along with their 95% CI are reflected as a diamond. All the analyses were conducted with the STATA Software version 13.0 (Stata Corporation, College Station, TX, USA).

Abbreviations for all figures: N: number of individuals; ES: effect size; CI: confidence intervals

**FigureS3a**: carotid Aix (adjusted for age, sex, BP) – cSVD


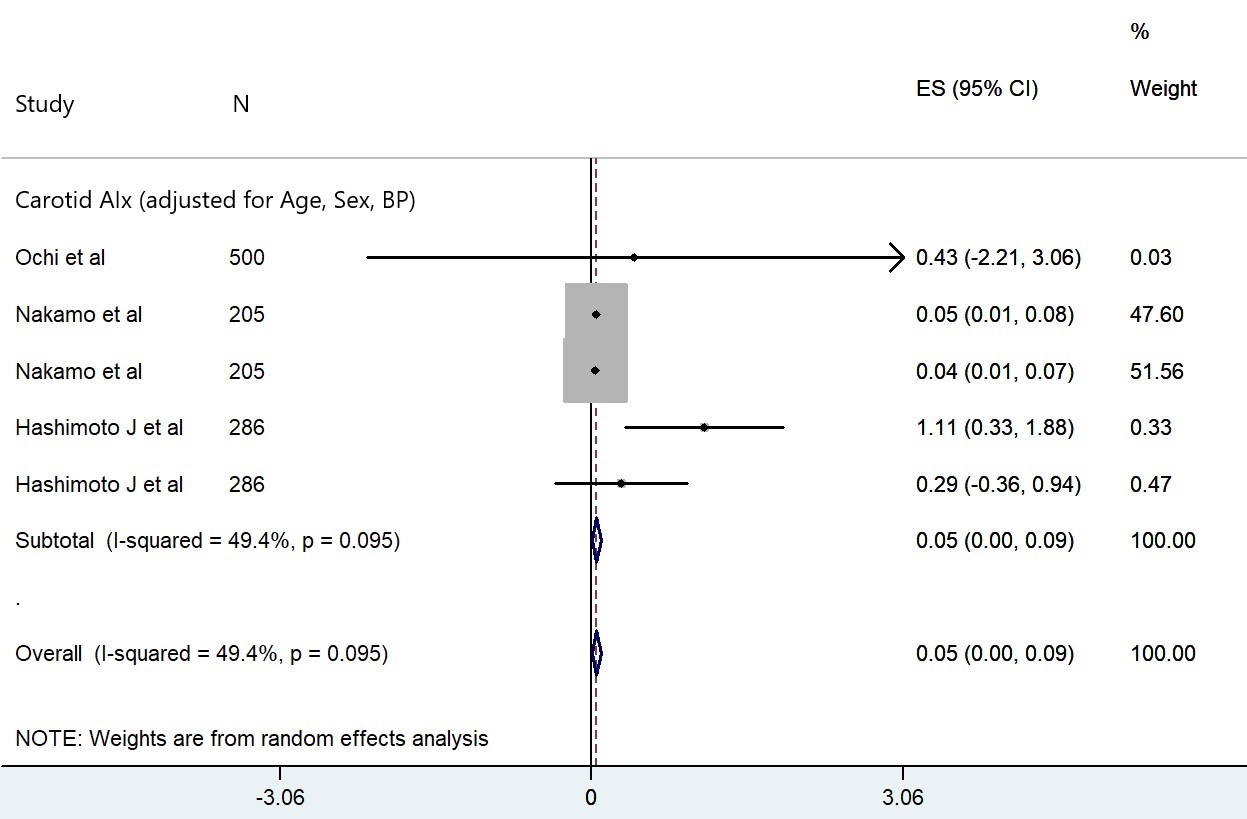


**FigureS3b**: carotid Aix (**with** flow Aix studies) – cSVD


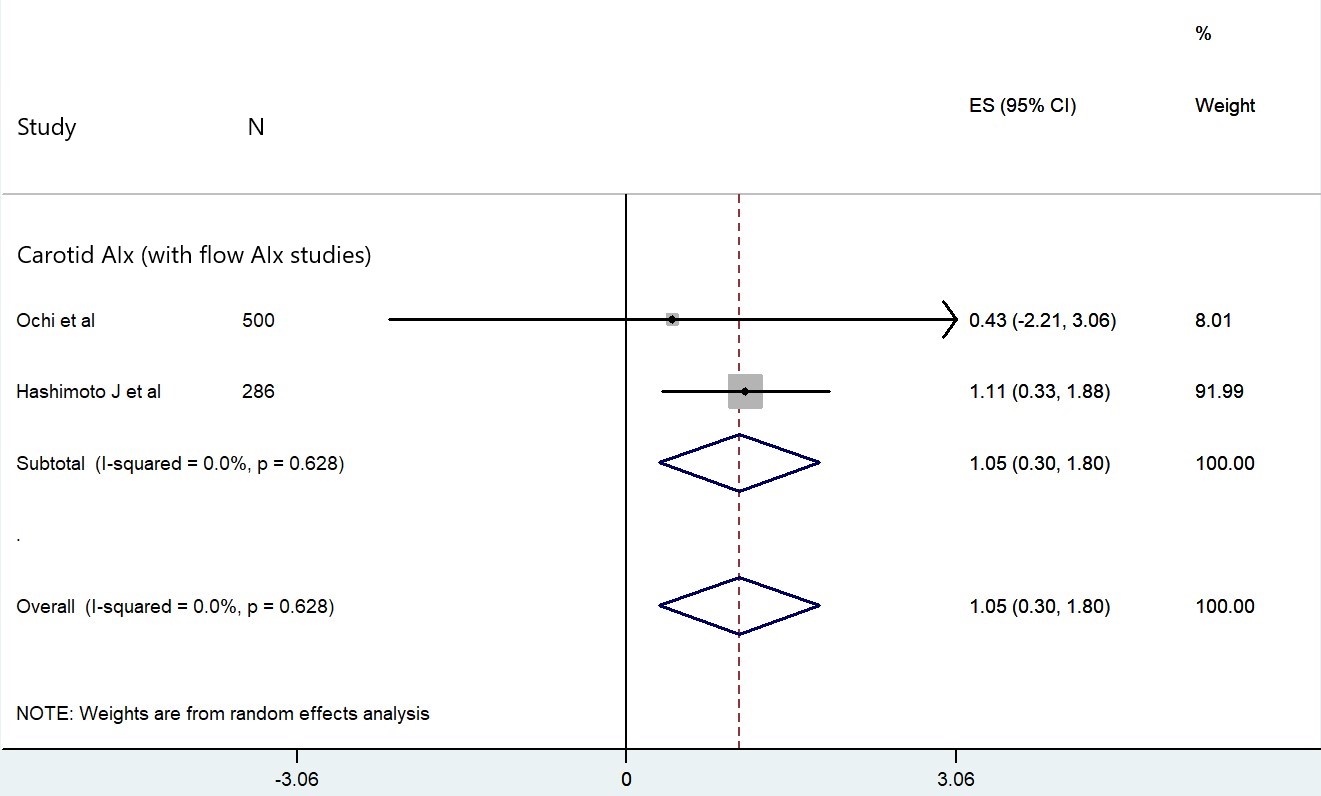


**Figure S4.** Forest plot of the meta-analysis association estimates between aortic AIx and cSVD in:

**(S4a) studies that used** adjusted models (at least for age, blood pressure, sex and heart rate),

In all figures, standardized beta coefficients (β) of each study are depicted as data markers; shaded boxes around the data markers indicate the statistical weight of the respective study; 95% confidence intervals (CI) are indicated by the error bars; pooled-effect estimates along with their 95% CI are reflected as a diamond. All the analyses were conducted with the STATA Software version 13.0 (Stata Corporation, College Station, TX, USA).

Abbreviations for all figures: N: number of individuals; ES: effect size; CI: confidence intervals

**Figure S4a**: aortic Aix (adjusted for age, sex, BP, HR) – cSVD

**
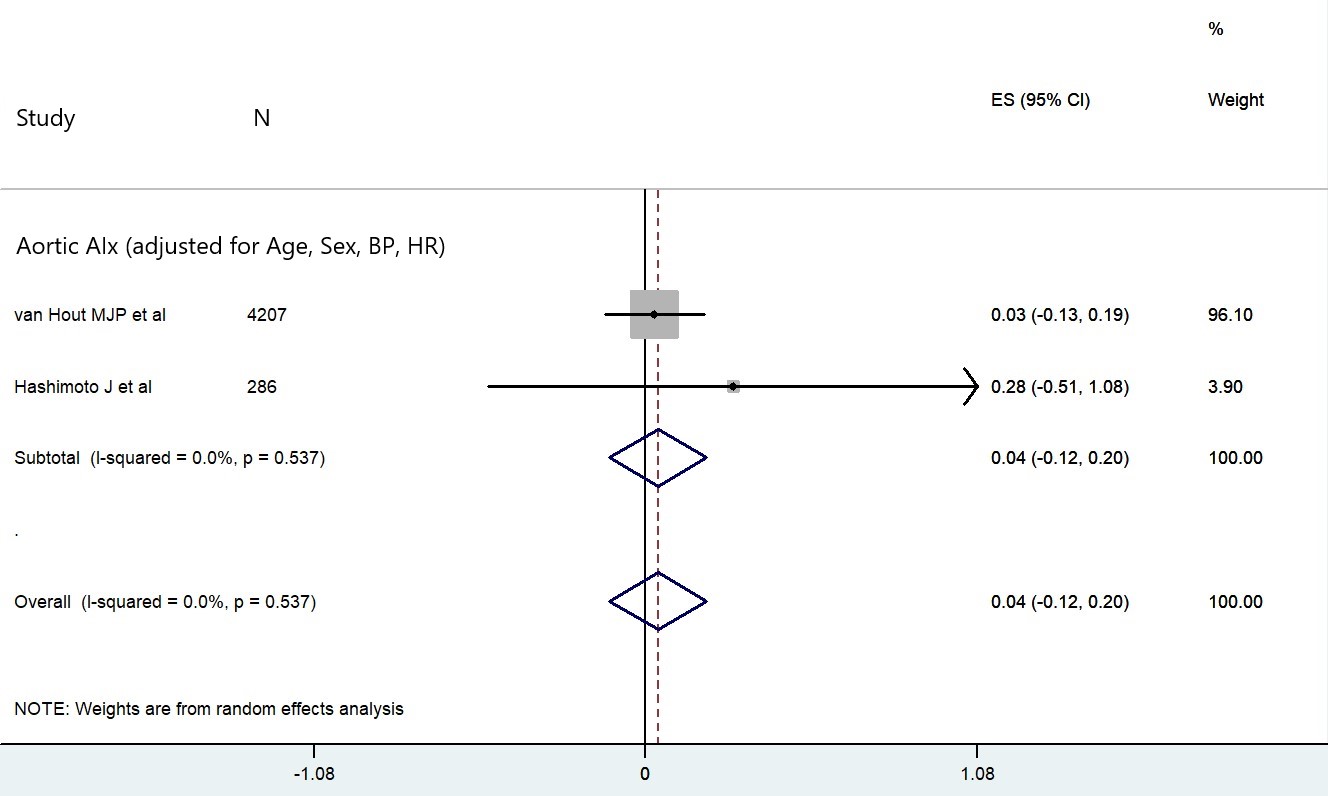
**

**Figure S5.** Forest plot of the meta-analysis association estimates between:

**(S5a)** central Aix and WMH in **all studies after excluding** flow Aix

**(S5b)** carotid Aix and WMH **all studies after excluding** flow Aix

In all figures, standardized beta coefficients (β) of each study are depicted as data markers; shaded boxes around the data markers indicate the statistical weight of the respective study; 95% confidence intervals (CI) are indicated by the error bars; pooled-effect estimates along with their 95% CI are reflected as a diamond. All the analyses were conducted with the STATA Software version 13.0 (Stata Corporation, College Station, TX, USA).

Abbreviations for all figures: N: number of individuals; ES: effect size; CI: confidence intervals

**Figure S5a**: central Aix (without flow Aix studies) – WMH


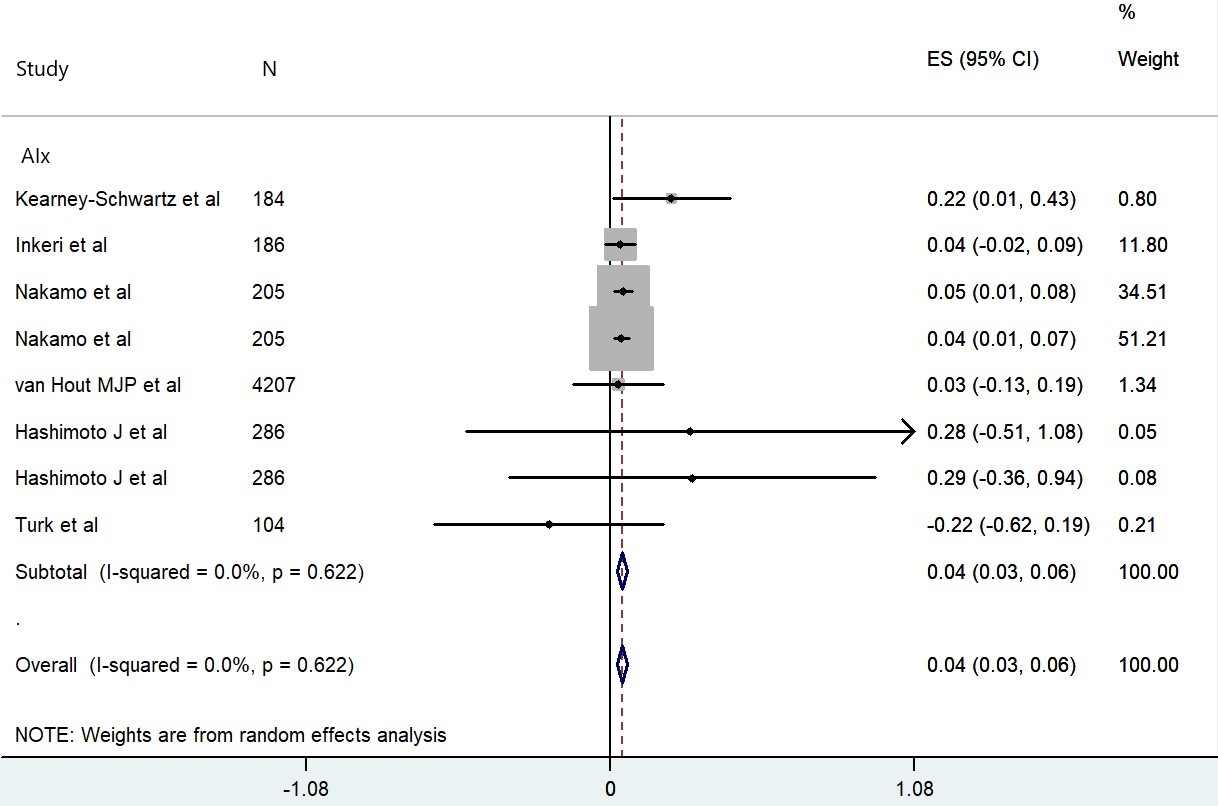


**Figure S5b**: carotid Aix (without flow Aix studies) – WMH


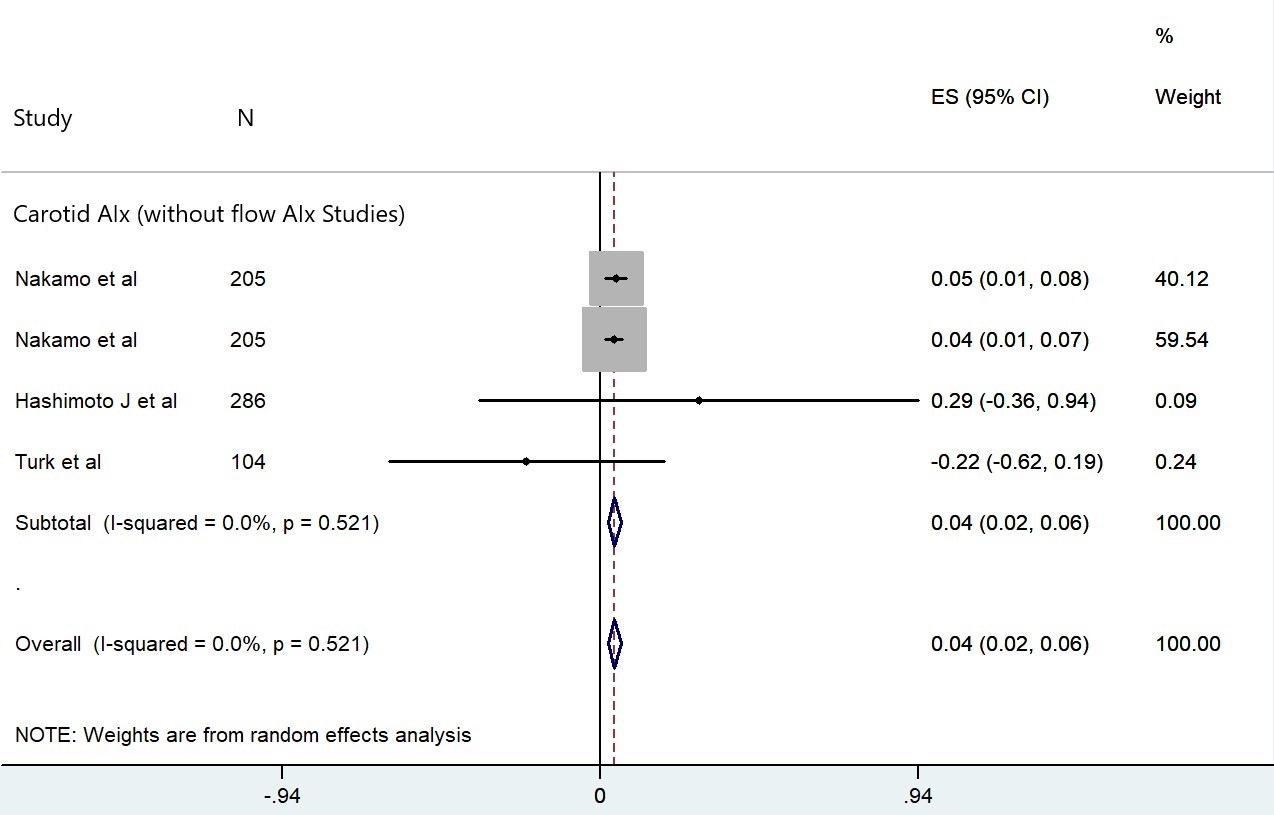

Supplement: hpaf054_suppl_Supplementary_Tables_S1-S3_Figures_S1-S51 [file hpaf054_suppl_supplementary_tables_s1-s3_figures_s1-s51.docx]
